# Baihui Point Laser Acupuncture Ameliorates Cognitive Impairment, Motor Deficit, and Neuronal Loss Partly via Antioxidant and Anti-Inflammatory Effects in an Animal Model of Focal Ischemic Stroke

**DOI:** 10.1155/2019/1204709

**Published:** 2019-02-24

**Authors:** Jinatta Jittiwat

**Affiliations:** ^1^Faculty of Medicine, Mahasarakham University, Mahasarakham, Thailand; ^2^Integrative Complimentary Alternative Medicine Research and Development Group, Khon Kaen University, Khon Kaen, Thailand

## Abstract

Stroke is recognized as one of the most dangerous killer diseases in Thailand and other countries worldwide. The development of a novel strategy for treating stroke patients is therefore urgently required. The present study investigated the effect of laser acupuncture at the Baihui point on cognitive and functional recovery, neuronal loss, antioxidant enzyme activities, and interleukin-6 (IL-6) activity in the hippocampus in an animal model of focal ischemic stroke. Male Wistar rats were randomly divided into 4 groups: sham operation; permanent occlusion of the right middle cerebral artery (Rt.MCAO); Rt.MCAO with sham laser acupuncture; and Rt.MCAO with Baihui point laser acupuncture. Laser acupuncture at the Baihui point and sham acupuncture at a nonacupoint were performed once daily (10 min at each point) for 14 days after Rt.MCAO. Half of the rats in each group were examined to determine neuron density by Cresyl violet staining, while the other half were examined by biochemical assays to measure glutathione peroxidase (GSH-Px) in the hippocampus, superoxide dismutase (SOD) in mitochondria and interleukin-6 (IL-6) activity in the hippocampus. Laser acupuncture treatment was found to significantly enhance memory and neuron density in CA1 and CA3. Improved neurological score, improved GSH-Px and SOD activities, and decreased density ratio of IL-6 to *β*-actin were also observed in the hippocampus. In conclusion, Baihui point laser acupuncture alleviates cognitive impairment and motor deficits via antioxidant and anti-inflammatory effects in focal ischemic rats. Further study is warranted to investigate other possible mechanisms of action.

## 1. Introduction

Ischemic stroke is characterized by an inadequate cerebral blood supply, and it has been recognized as one of the most dangerous killer diseases in Thailand [1] and other countries worldwide [2], especially developing countries [3]. Stroke is a common cause of neurological problems such as cognitive impairment and motor and sensory deficit that is mainly found in the hospital and usually leads to mortality or disability problems [4]. In cerebral ischemia, free radical generation dramatically increases and overwhelms endogenous antioxidant systems, leading to a breakdown in equilibrium [5]. Additionally, it has been shown that inflammation plays a crucial role in both the pathogenesis and prognosis of ischemic stroke [6]. The prognosis for stroke patients with inflammation appears to be very poor, and the inhibition of inflammation could decrease brain infarct size and improve neurological deficit in experimental stroke [7]. Several studies also demonstrate that neurotoxicity induced by inflammation and stroke is associated with elevation of interleukin-6 (IL-6) including one study showing that IL-6 correlates significantly with the outcome and mortality rate of ischemic stroke patients [8].

Laser acupuncture is a noninvasive intervention, which applies low level laser therapy (LLLT) to an acupuncture point. Considerable research has been published to show that laser acupuncture can be used to treat insomnia, Alzheimer's disease, autism, psychiatric disorders, spinal cord injury, Parkinson's disease, and stroke [9–12]. In addition, numerous studies have demonstrated that LLLT at a wavelength of 810 nm can improve neurological deficit and protect against brain neuronal damage [13, 14]. The physiological effects of LLLT are reported to include vasodilation, increased cell proliferation, increased production of anti-inflammatory metabolites, reduced edema, increased oxygen availability, and stimulation of mitochondrial activity [15–17]. Moreover, traditional acupuncture at the Baihui point (Governing vessel; GV20) is widely used to treat clinical disorders such as paralysis, dizziness, headache, poor memory, and stroke [12, 18]. Thus, this study was carried out to investigate the effects of laser acupuncture at the Baihui point on cognitive and functional recovery, neuronal loss, antioxidant enzyme activity, and IL-6 activity in the hippocampus in an animal model of focal ischemic stroke induced by permanent occlusion of the right middle cerebral artery (Rt.MCAO).

## 2. Methods

### 2.1. Chemicals and Antibody

Digitonin, sucrose, cresyl violet, glutathione reductase (GR), *β*-nicotinamide adenine dinucleotide phosphate (NADPH), D-mannitol,* t*-butyl hydroperoxide, ethylenediaminetetraacetic acid (EDTA), bovine serum albumin (BSA), ethylene glycol-bis (2-aminoethylether)-*N,N,N*′*,N*′-tetraacetic acid (EGTA), HEPES, sodium orthovanadate, protease inhibitor cocktail, and anti-mouse IgG peroxidase conjugated secondary antibody were purchased from Sigma-Aldrich (St. Louis, MO, USA). Tris hydrochloride (Tris-HCl), Triton x-100, tween-20, and sodium dodecyl sulfate (SDS) were purchased from Bio-Rad Laboratories, Inc. (Hercules, California, USA). Mouse monoclonal anti-IL-6 antibody was purchased from Abcam (Abcam, MA, USA).

### 2.2. Animals

Male Wistar rats weighing 300-350 g (8 weeks old) were purchased from the National Laboratory Animal Center (Salaya, Nakhon Pathom, Thailand). Rats were housed in groups of 5 per cage in standard metal cages and under restriction controlled temperature at 23±2°C and a 12:12 h light-dark cycle. Water and food were freely available to all rats (24hr/day). All experimental techniques were designed and carried out in accordance with the directives for laboratory use and care of animals, issued by the Institutional Animal Care and Use Committee, Khon Kaen University, Thailand (Record No. AEKKU 28/2558).

### 2.3. Animal Treatment

The rats were randomly divided into the following 4 groups (n=10 per group):

  Group I: Sham operation group (Sham). Rats were given a sham operation with no treatment.
  Group II: Rt.MCAO group (Rt.MCAO). All rats in this group were induced with a focal cerebral ischemia by a right middle cerebral artery occlusion (Rt.MCAO) with no treatment.
  Group III: Rt.MCAO with sham laser acupuncture group (Sham laser acupuncture). The rats were induced with a focal cerebral ischemia by Rt.MCAO, with subsequent laser acupuncture at a nonacupoint located 5 mm next to the Baihui point.
  Group IV: Rt.MCAO with laser acupuncture group (laser acupuncture). Rats were induced with a focal cerebral ischemia by Rt.MCAO, with subsequent laser acupuncture at the Baihui point.

All animals were treated with the assigned intervention once daily for 14 days after the occlusion of the right middle cerebral artery. Spatial memory was determined using the Morris water maze test, while recovery of motor function was assessed by neurological score grading 1 day, 7 days, and 14 days after Rt.MCAO. The density of neurons in the hippocampus was determined at the end of the experiment. The glutathione peroxidase (GSH-Px) and superoxide dismutase (SOD) activities, and the IL-6 expression in the rat hippocampus were also determined using several biochemical assays at the end of the experiment.

### 2.4. Induction of Focal Cerebral Ischemia

All experimental animals were fasted for 12 h before surgery, but they were allowed free access to water. The rats were anesthetized with an intraperitoneal injection of pentobarbital sodium at a dose of 40 mg/kg body weight. Focal cerebral ischemia was induced by permanent intraluminal occlusion of the right middle cerebral artery [19]. In brief, the right common carotid artery and the right external carotid artery were exposed through a ventral midline neck incision and were proximally ligated. A silicone coated round-tipped nylon monofilament (4-0) suture (the USS DG™ division of United States Surgical; Tyco Healthcare Group LP, Norwalk, CT, USA) was inserted into the common carotid artery just below the carotid bifurcation and advanced into the internal carotid artery approximately 17-19 mm distal to the carotid bifurcation until a slight resistance was felt. Finally, the right middle cerebral artery was occluded by the tip. The wound was sutured and 10% povidone iodine solution was infiltrated at the incision sites for antiseptic postoperative care. All the operated rats were transferred back to their cages without food or water restriction. For the sham surgery, all arteries were exposed as above but the filament was not inserted into the middle cerebral artery.

### 2.5. Laser Acupuncture

To avoid stressing the rats before laser acupuncture treatment, rats were anesthetized with pentobarbital sodium at a dose of 40 mg/kg body weight. The rats were treated with laser acupuncture at the Baihui acupoint and a nonacupoint (10 min at each point) once daily for 14 days. The 810 nm laser beam delivered a laser spot diameter of 100 *μ*m at the acupoint and nonacupoint (Weberneedle® Lauenförde, Germany) with a laser module output of 100 mW, as pulsed waves (50% duty cycle).

### 2.6. Determination of Cognitive Function

Cognitive function was determined using the Morris water maze test as described previously [20]. In brief, the water maze composed of a metal pool 170 cm in diameter and 58 cm deep, filled with tap water at room temperature, and divided into 4 quadrants (Northeast, Northwest, Southeast, and Southwest). A removable escape platform was placed in the center of 1 quadrant, 2 cm below the water level, and was covered with a nontoxic milk powder. In training sessions, if the animal could not reach the platform within 60 s, it was gently guided to the platform and left there for 15 s before removing it from the pool. The time taken for the animal to climb onto the hidden platform was recorded as escape latency. Retention memory was determined 24 h later by removing the platform and placing the animals into the water maze for 60 s. The retention time was recorded as the time taken for a rat to swim to the previous location.

### 2.7. Determination of Neurological Score

Assessment of neurological scores was performed by a single experimenter, who was blinded to the experimental groups according to the method of Bederson et al. [21], with a modification as described in our previous study [22]. The deficit was graded with a scale ranging from 0-5, with higher scores representing better neurological function.

### 2.8. Cresyl Violet Staining for Nissl Substance and Morphology Analysis

Adjacent series of sections of the hippocampus from all experimental groups were stained with 0.5% cresyl violet to aid in neuronal density determination. Regions of the hippocampus (CA1 and CA3) were evaluated using a Zeiss light microscope, model ZEISS AxioCam ICc 3 (Carl Zeiss Microscopy GmbH, Jena, Germany). To determine the neuronal density, five coronal sections containing hippocampus were selected for analysis at stereotaxic coordinates as described in our previous publication [23]. The observer who analyzed the sections was not informed of the treatment at the time of analysis. The density of surviving neurons was determined at 40x magnification and the data was presented as % of sham.

### 2.9. Determination of Anti-Oxidative Markers

Rats were perfused with cold phosphate buffered saline to remove blood from the brain tissue. Brain tissue was then immediately stored at −80°C until used. The hippocampus was separated and homogenized in a buffer (10 mM sucrose, 10 mM Tris-HCl, 0.1 mM EDTA, and adjusted to pH 7.4).

### 2.10. Protein Determination

The protein concentration in the above brain homogenate samples was measured using the method of Lowry and colleagues [24], and BSA was used as a standard. The quantity of protein in the hippocampus sample was spectrophotometrically recorded at 650 nm.

### 2.11. Glutathione Peroxidase (GSH-Px) Level

GSH-Px activity in the hippocampi was determined spectrophotometrically at 340 nm with activity determined using* t*-butyl hydroperoxide as a substrate and GR and NADPH as enzymatic and nonenzymatic indicators, respectively [25]. The data were expressed as units/mg protein.

### 2.12. Isolation of Mitochondria from Hippocampus for Determination of Superoxide Dismutase (SOD) Activity

Rats were sacrificed and their right hippocampus was removed at the end of the experiment. These were homogenized in mitochondrial isolation buffer containing 225 mM mannitol, 75 mM sucrose, 1 mM EGTA, 20 mM HEPES, 0.1% BSA, and 0.01% digitonin adjusted to pH 7.2 with KOH. The tissue homogenates were centrifuged at 1,000 g for 2 minutes at 4°C. Supernatants were transferred into clean 1.7 ml Eppendorf® tubes, and pellets were resuspended in 0.2 ml of isolation buffer and centrifuged again at 1,000 g for 2 min. Both supernatants were combined and mixed with 0.07 ml of 80 vol% Percoll solution: 1 M sucrose, 50 mM HEPES, and 10 mM EGTA adjusted to pH 7.0 with KOH. A volume of 0.7 ml of 10% Percoll solution was gently layered on top, and the mixture was centrifuged for 10 min at 18,500 g. The 10 vol% Percoll solution was prepared by diluting 80 vol% Percoll solution with isolation buffer. The mitochondrial pellet was further purified by resuspending in 0.7 ml of washing buffer (250 mM sucrose, 5 mM HEPES, 0.1 mM EGTA, and 1 mg/ml of BSA adjusted to pH 7.2 with KOH) and centrifuging for 5 min at 10,000 g. Thereafter, the final mitochondrial pellet was suspended in 0.07 ml of washing buffer and stored at −80°C until used [26]. SOD activity was determined using an SOD assay kit (Sigma-Aldrich) according to the manufacturer's instructions.

### 2.13. Determination of Interleukin-6 (IL-6)

At the end of the experiment, rats were sacrificed and their right hippocampus was removed. Each hippocampus was homogenized in 10 volumes of ice cold lysis buffer (150 mM sodium chloride, 50 mM Tris–hydrochloride, 0.25 mM EDTA, 1% Triton X-100, 0.1% sodium orthovanadate, and 0.1% protease inhibitor cocktail, pH 7.4). The homogenate was further centrifuged at 4°C at a speed of 12,000 g for 30 min. After the centrifugation, supernatant was collected, resolved in 10% SDS–polyacrylamide gel under reducing conditions, and electrotransferred to polyvinylidene difluoride (PVDF) membrane (Immobilon-P, Millipore, Bedford, MA, USA). Then, nonspecific binding sites on the membrane were blocked by incubating with 5% nonfat dried milk in 0.1% Tween-20 in Tris buffered saline (TBS-T), pH 7.4 for 3 h at room temperature. The membrane was incubated with mouse monoclonal anti-IL-6 antibody (1:2000) at 4°C overnight. After washing 3 times with TBS-T, the membrane was incubated with anti-mouse IgG peroxidase conjugated secondary antibody at room temperature for 1 hour. Immunoreactivity was visualized using a chemiluminescent substrate (SuperSignal West Pico, Pierce, Rockford, IL). The density of the IL-6 band was normalized against *β*-actin, and the mean ratio was calculated using the ChemiDoc™ MP imaging system with image lab software (Bio-Rad Laboratories Inc., Hercules, California, USA).

### 2.14. Statistical Analysis

The data were expressed as mean value ± the standard error of mean (S.E.M.). The statistical significance of the data was determined by one-way analysis of variance (ANOVA), followed by the post hoc least significant difference (LSD) paired comparisons test. A* p*-value < 0.05 was considered statistically significant. All data were processed with SPSS statistical software (SPSS-IBM Inc., Chicago, IL, USA).

## 3. Results

### 3.1. Effect of Baihui Point Laser Acupuncture on the Recovery of Cognitive Impairment and Motor Function

**Figure 1:**
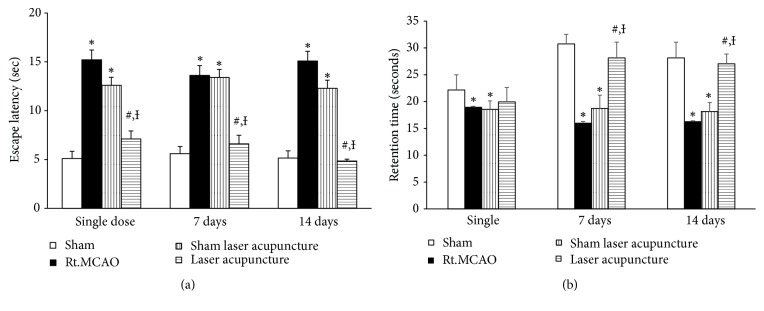
Effect of Baihui point laser acupuncture on escape latency (a) and retention time (b) in the Morris water maze test. The data are expressed as mean±S.E.M. (n=5). ^*∗*^*p*-value<0.05 when compared to the sham; ^**#**^*p*-value<0.05 when compared to the Rt.MCAO group; and ^**Ɨ**^*p*-value<0.05 when compared to the sham laser acupuncture group.

Based on the fact that cognitive impairment and contralateral paralysis usually develops after cerebral ischemia induced by middle cerebral artery occlusion, the present study also determined the effect of laser acupuncture on the functional recovery of cognitive impairment and motor function. It was demonstrated that the animals with focal cerebral ischemic stroke induced by permanent Rt.MCAO had significantly increased escape latency and decreased retention time compared to the sham group as shown in Figures 1(a) and 1(b). Escape latency was significantly decreased in animals treated with laser acupuncture treatment (p-value<0.05) throughout the experimental period. Significantly enhanced retention time was shown only at 7 and 14 days after laser acupuncture treatment (p-value<0.05) compared to the Rt.MCAO group and sham laser acupuncture group.

**Figure 2:**
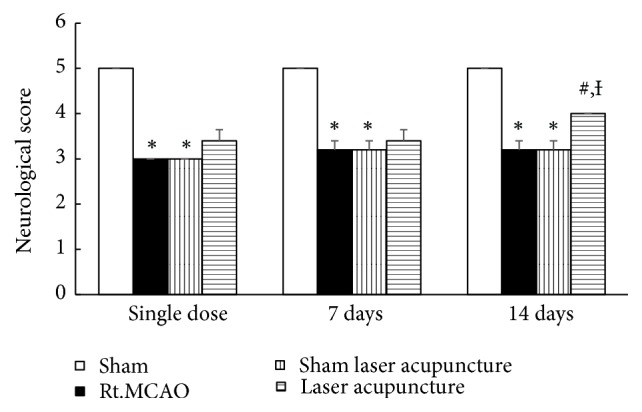
Effect of Baihui point laser acupuncture on the motor performance using neurological score in cerebral ischemic rats. The data are expressed as mean±S.E.M. (n=5). ^*∗*^*p*-value<0.05 when compared to the sham; ^**#**^*p*-value<0.05 when compared to the Rt.MCAO group; and ^**Ɨ**^*p*-value<0.05 when compared to the sham laser acupuncture group.

The effect of laser acupuncture at the Baihui point on neurological scores is shown in Figure 2. Animals with focal ischemic stroke induced by permanent Rt.MCAO had significantly decreased neurological scores compared to the sham group (p-value<0.05) throughout the 14-day experimental period. Interestingly, laser acupuncture significantly improved neurological scores at 14 days after treatment (p-value<0.05) compared to the Rt.MCAO group and sham laser acupuncture group.

### 3.2. Effect of Baihui Point Laser Acupuncture on Neuronal Density in the Hippocampus

**Figure 3:**
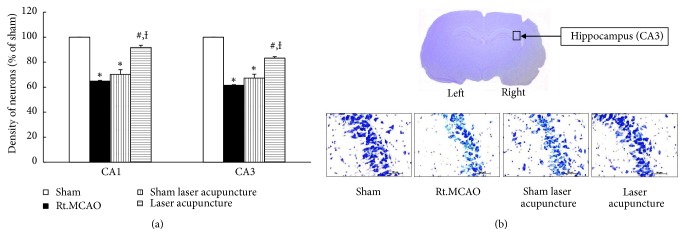
Effect of Baihui point laser acupuncture on neuronal density in the CA1 and CA3 regions of the hippocampus. (a) Neuronal density in CA1 and CA3 of the hippocampus. (b) Photograph of coronal sections of CA3 stained with cresyl violet at 40x magnification. The data are expressed as mean±S.E.M. (n=5). ^*∗*^*p*-value<0.05 when compared to the sham; ^**#**^*p*-value<0.05 when compared to the Rt.MCAO group; and ^**Ɨ**^*p*-value<0.05 when compared to the sham laser acupuncture group.

This study demonstrated that permanent Rt.MCAO significantly decreased neuronal density in CA1 and CA3 of the hippocampus. Sham laser acupuncture failed to alleviate the reduction of survival density in both CA1 and CA3, while laser acupuncture at the Baihui point significantly improved the decreased survival density induced by permanent Rt.MCAO in CA1 and CA3 (*p-*value<0.05; compared to Rt.MCAO group and sham laser acupuncture group) as shown in Figure 3.

### 3.3. Effect of Baihui Point Laser Acupuncture on Antioxidant Enzymes in Hippocampus

**Figure 4:**
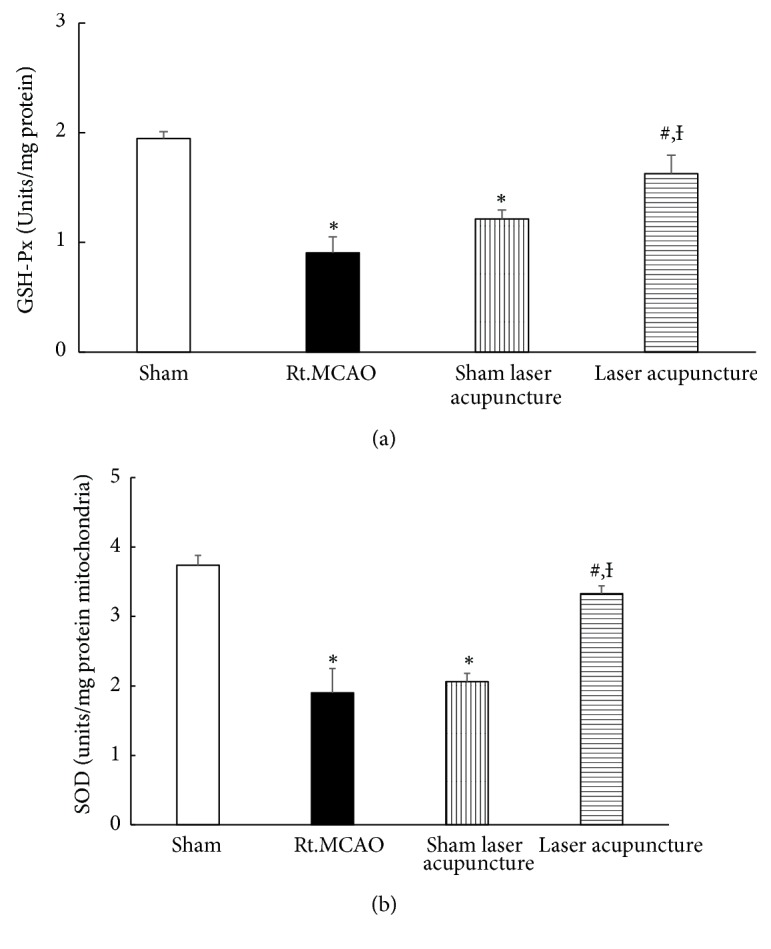
Effect of Baihui point laser acupuncture on antioxidant enzymes. (a) Glutathione peroxidase (GSH-Px) activity in the hippocampus. (b) Superoxide dismutase (SOD) activity in mitochondria in the hippocampus. The data are expressed as mean±S.E.M. (n=5). ^*∗*^*p*-value<0.05 when compared to the sham; ^**#**^*p*-value<0.05 when compared to the Rt.MCAO group; and ^**Ɨ**^*p*-value<0.05 when compared to the sham laser acupuncture group.

The present study found that cerebral ischemic rats had significantly decreased GSH-Px activity in the hippocampus area (p-value<0.05 compared to the sham group). Sham laser acupuncture did not induce significant changes in GSH-Px activity, whereas laser acupuncture at the Baihui point improved GSH-Px activity compared to the Rt.MCAO group and sham laser acupuncture group (p-value<0.05) as shown in Figure 4(a). Moreover, permanent Rt.MCAO induced a significant decrease in mitochondrial SOD activity in the hippocampus area (p-value<0.05 compared to the sham group), as shown in Figure 4(b). In laser acupuncture treated rats, the mitochondrial SOD activity was significantly higher in the hippocampus compared to the Rt.MCAO group and sham laser acupuncture group (p-value<0.05).

### 3.4. Effect of Baihui Point Laser Acupuncture on Western Blot Analysis of IL-6

**Figure 5:**
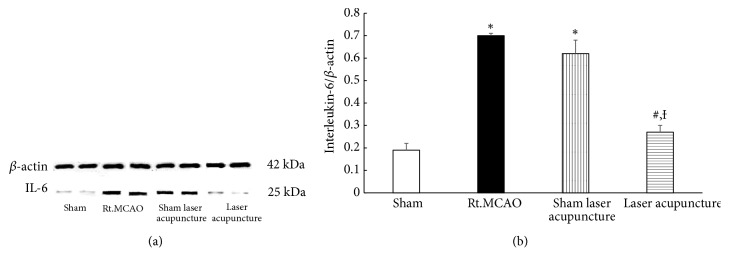
Western blot analysis of homogenates of the hippocampus for sham, MCAO, sham laser acupuncture, and laser acupuncture groups. (a) Antibody to IL-6 detected as a single band at 25 kDa. (b) Quantitative western blot. The IL-6 band was normalized with beta-actin. The data are expressed as mean±S.E.M. (n=5). ^*∗*^*p*-value<0.05 when compared to the sham; ^**#**^*p*-value<0.05 when compared to the Rt.MCAO group; and ^**Ɨ**^*p*-value<0.05 when compared to the sham laser acupuncture group.

The determination of IL-6 level in the hippocampus was performed by western blot analysis. The bands which showed positive immunoreactivities against *β*-actin and IL-6 were detected at 42 kDa and 25 kDa, respectively. A significant decrease in the density ratio of IL-6 to *β*-actin band was observed in cerebral ischemic rats subjected to laser acupuncture at the 14-day study period as shown in Figure 5 (*p-*value<0.05; compared to Rt.MCAO group and sham laser acupuncture group).

## 4. Discussion

Ischemic stroke represents a common cause of dementia, reduced cognitive function, and neurological deficits in later years either alone or in concurrence with other pathologies [27, 28]. This study used the permanent occlusion of the right middle cerebral artery model (Rt.MCAO) to investigate an experimental therapy. The Rt.MCAO model generates motor and cognitive function deficits and significant impairments in the hippocampus as previously reported [29, 30]. The present study determined the effect of laser acupuncture on learning memory and motor function in a cerebral ischemic condition induced by Rt.MCAO. Laser acupuncture was shown to attenuate memory impairment and facilitate the recovery of motor function induced by permanent Rt.MCAO.

Previous studies have shown that patients with cerebral ischemic stroke have a greatly reduced number of neurons in brain regions such as the cerebral cortex and hippocampus [31], and that a correlation exists between cerebral ischemia-induced hippocampal damage and spatial learning deficit [32]. Therefore, the present study investigated the effect of laser acupuncture on neuron density in the hippocampus. The results show that laser acupuncture at the Baihui point significantly improves the low survival density induced by permanent Rt.MCAO in the CA1 and CA3 regions of the hippocampus. A previous study found that the hippocampal CA1 region is involved in cognitive function and contains a diverse range of neurons important for information processing. Additionally, CA3 contains abundant glutamate receptors, particularly NMDA receptors, which play an important role in associative memory [33]. Moreover, they also play an important role in encoding new spatial information within short-term memory [34]. A considerable body of evidence now suggests that laser acupuncture stimulation increases CREB activity and reduces neuronal cell loss in the hippocampus [35, 36], which can improve memory impairment in various disorders including Alzheimer's disease, Parkinson's disease, and stroke [11, 36, 37]. Traditional acupuncture at the Baihui point (Governing vessel; GV20) is widely used to treat clinical disorders such as paralysis, dizziness, headache, poor memory, and stroke [12, 18]. The present study suggests a possible mechanism by which laser acupuncture at the Baihui point might attenuate memory impairment in cerebral ischemia—the improved survival of neurons in CA1 and CA3.

Reactive oxygen species (ROS) and lipid peroxidation (LPO) have been proposed to be important factors in reducing cerebral blood flow and reperfusion injury [38]. It has also been postulated that neurodegeneration is associated with ROS, which react with cellular macromolecules such as lipids, proteins, and nucleic acids leading to neuron damage [39]. The endogenous antioxidant enzyme activity of the ischemic brain is therefore very important, and the measurement of antioxidant enzymes could be used as a tool to assess the vulnerability of areas of the ischemic brain [40]. The present study showed that Rt.MCAO could decrease the activities of GSH-Px and SOD. Given the previous finding that laser acupuncture has an antioxidant effect [41], this represents another possible mechanism by which laser acupuncture could be of benefit following cerebral ischemia. ROS can be generated within mitochondria in cerebral ischemic cascade, while 810 nm laser therapy is able to reduce markers of oxidative stress and increase the activity of antioxidant enzymes such as glutathione (GSH), glutathione reductase (GR), and superoxide dismutase (SOD) [12, 17, 41]. Furthermore, 810 nm laser has been shown to increase mitochondrial activity and ATP synthesis, which could also improve neurological deficits after stroke [42].

The prognosis for stroke patients with inflammation appears to be very poor, and inhibition of inflammation can decrease brain infarct size and improve neurological deficit in experimental stroke [7]. Several lines of evidence demonstrate that stroke- and inflammation-induced neurotoxicity is associated with elevated IL-6. Also, previous studies found a significant correlation between IL-6 levels and the outcome and mortality rate of ischemic stroke patients [8]. The results from this study showed that there was a significant decrease in the density ratio of IL-6 to *β*-actin band in cerebral ischemic rats subjected to laser acupuncture for 14 days. Previous studies have also confirmed the effectiveness of laser acupuncture in alleviating inflammation [43] because of its efficacy in inhibiting most of the proinflammatory cytokines.

## 5. Summary and Conclusion

**Figure 6:**
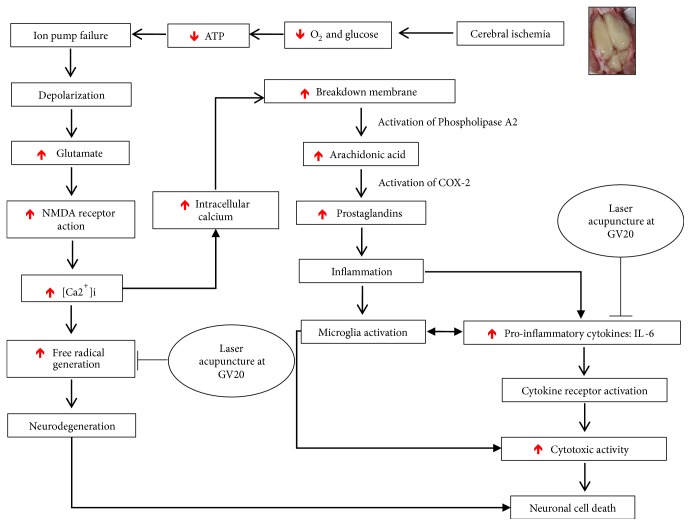
Possible mechanism of action of Baihui point laser acupuncture.

In cerebral ischemia, energy failure causes depolarization of the neuronal membrane, and excitatory neurotransmitters such as glutamate are released. A marked influx of Ca^2+^ into neurons then occurs, which provokes an enzymatic process leading to irreversible neuronal injury [44]. Free radical generation also dramatically increases and overwhelms endogenous antioxidant systems, leading to a breakdown of equilibrium [5] and inflammation, being a contributing factor in the development of ischemic damage [6]. Baihui point laser acupuncture has antioxidant and anti-inflammatory effects (Figure 6), which can improve neuronal loss in the hippocampus and cognitive and motor impairments. Further study is now warranted to investigate other possible mechanisms by which Baihui point laser acupuncture could affect cognitive and motor function in focal ischemic stroke.

## Data Availability

The data used to support the findings of this study are available from the corresponding author upon request.
